# M2 Tumor Associate Macrophage- (TAM-) Derived lncRNA HISLA Promotes EMT Potential in Bladder Cancer

**DOI:** 10.1155/2022/8268719

**Published:** 2022-05-06

**Authors:** Yuanyuan Guo, Zhong Li, Wei Sun, Wuyue Gao, Yujie Liang, Zhijie Mei, Beibei Liu, Rui Wang

**Affiliations:** ^1^Department of Urology, The First Affiliated Hospital of Bengbu Medical College, Bengbu City, Anhui Province 233004, China; ^2^Department of Oncology, The First Affiliated Hospital of Bengbu Medical College, Bengbu City, Anhui Province 233004, China

## Abstract

**Background:**

Tumor-associated macrophages (TAMs) are M2-like phenotype macrophages which contribute to the tumor progression in tumor microenvironment. The precise mechanisms of TAMs were intricated, and recently, it has been illustrated that TAM-derived exosomal lncRNAs played pivotal roles in the tumor development. In the present study, we investigated the role of TAM-derived exosomal lncRNA HISLA in bladder cancer.

**Materials and Methods:**

Effects of TAM exosomes and exosomal lncRNA HISLA on migration and invasion in bladder cells were detected by wound healing assay, transwell assay, and western blot assay. Differential expression of lncRNA HISLA in exosomes derived from M0 or TAMs was examined by qRT-PCR. Western blot assay was used to classify the precise molecular mechanisms.

**Results:**

We found that TAM-derived exosomes significantly promote the migration and invasion abilities of bladder cells. Expression of epithelial-mesenchymal transition (EMT) markers was obviously affected by TAM exosome administration. Furthermore, we found that the expression of lncRNA HISLA was specifically elevated in TAM exosomes and TAM exosome-treated bladder cells. Silencing of lncRNA HISLA was found to suppress the processes of migration, invasion, and EMT in bladder cells. In addition, we found that *β*-catenin levels were downregulated, and Ser33 phosphorylated *β*-catenin levels were increased by HISLA siRNA treatment. At last, we found that HISLA stabilized *β*-catenin expression through preventing interaction between GSK3*β* and *β*-catenin.

**Conclusion:**

In conclusion, our results investigated the prometastatic role of exosomal lncRNA HISLA derived from TAMs in bladder cancer and suggested TAM-derived HISLA as a promising therapeutic target of bladder cancer.

## 1. Introduction

Bladder cancer (BC) causes nearly 170000 deaths worldwide annually, and it accounts for the first incidence of genitourinary cancer in China [1]. Currently, the treatment of bladder cancer is still mainly based on surgery, radiotherapy, and chemotherapy, and although the progress of surgery, chemotherapy, and molecular-targeted drugs has been developed, the prognosis of BC patients was not significantly improved which mainly due to the prone recurrence, invasion, and metastasis characteristics of BC [2, 3]. Therefore, investigating the mechanism of invasion and metastasis of bladder cancer is crucial for the treatment of bladder cancer.

Tumor-associated macrophages (TAMs) refer to the macrophages that infiltrate into the tumor microenvironment (TME) [4]. Typically, TAMs mainly polarized to M2-like macrophages which act as accomplice in malignant tumor progression [5]. For instance, CCL2-CCR2 axis was reported to induce immune evasion through PD-1 signaling in esophageal carcinogenesis via recruiting AMs [6]. TAMs contributed to ovarian cancer cell migration through secreting TGFBI and tenascin C [7]. TAM-derived CCL5 was shown to promote prostate cancer stem cells and metastasis via activating *β*-catenin/STAT3 signaling [8]. Furthermore, researches revealed that TAM-derived exosomes promoted the migration of gastric cancer cells by transfer of functional ApoE [9]. These findings suggested M2-TAMs as potential targets for the tumor treatment especially through regulating metastasis process.

Exosomes were secreted organelles with single membrane, which has diameter of ~100 nanometers and containing of great varieties of specific nucleic acids, lipids, and proteins [10]. As the most important vesicular transport structure between cells, exosomes have been found to play critical roles in cell communication as well as various physiological activities [11]. It has been proved that exosomes participated almost all the processes of tumor and serve essential roles in communication within the tumor microenvironment [12]. Long noncoding RNAs (lncRNAs) have been proved to be one of the most numerous communication media transmitted by exosomes and contribute to the regulation of cell proliferation, apoptosis, metastasis, autophagy, macrophage polarization, and other phenomenon of different kinds of cancers [13–15]. In bladder cancer, exosome-transmitted lncRNA PTENP1 was reported to suppress bladder cancer progression by competitively binding to microRNA-17 [16], exosomal lncRNA LNMAT2 illustrated to promote lymphatic metastasis in bladder cancer [17], and hypoxic exosomes were found to facilitate bladder tumor growth and development through transferring lncRNA UCA1 [18].

In the current manuscript, we revealed that lncRNA HISLA derived from TAMs contributed to enhance the EMT potential of BC by stabilizing *β*-catenin expression, and our findings suggested HISLA as a potential medical target for the bladder cancer treatment.

## 2. Materials and Methods

### 2.1. Cell Culture and M2-Like TAM Polarization

Human bladder cancer cell lines T24 and HTB-1 and human monocyte cell line THP-1 were all obtained from the Chinese Academy of Science (Shanghai, China). All cells were maintained in DMEM culture medium containing 10% FBS (Gibco), 100 U/ml penicillin, and 100 *μ*g/ml streptomycin at 37°C supplied with 5% CO_2_ atmosphere. For M2-like TAM polarization, M0 THP-1 cells were incubated with IL-4 and IL-13 (R&D Systems) at 20 ng/ml concentration for 24 hours, and the markers of M2-TAMs including IL-10, CCL-18, CD206, CD163, Clever-1, and Arg-1 were detected.

### 2.2. Exosome Isolation and Identification

After reached 80% confluence, the polarized macrophages were incubated for 48 h in complete DMEM medium with 10% exosome-depleted FBS, and exosomes were isolated from the medium by differential centrifugation as described [19]. For identification of exosomes, transmission electron microscopy (TEM) and Nano-LC–MS/MS analysis were also performed as previously reported [9]. The purified exosomes were resuspended in PBS followed by cell treatment or RNA extraction. The PKH67 Green Fluorescent Cell Linker Kit (Sigma) was used for exosomes labelling according to the manufacturer's protocol. To examine the exosomal uptake into BC cells, the T24 cells were grown in 24-well plates and incubated with PKH67-labelled exosomes from polarized M2-like TAMs. DAPI was used to stain the nuclei, and fluorescence microscope (Zeiss, LSM700B, Germany) was used for visualization of immunofluorescence.

### 2.3. Wound Healing Assay

After treatment with si-Control or si-HISLA exosomes for 24 hours, BC cells were seeded into 6-well plates, and when cells were grown to 80–90% confluence, a scratch wound was made by using a 200 *μ*l plastic pipette tip. The floating cells were washed three times by PBS. The scratches were photographed after 24 h by using microscopy.

### 2.4. Migration and Invasion Assays

Cell migration and invasion assays were conducted using Transwell Permeable Supports (Corning, NY, USA), and matrigel (Corning, NY, USA) was coated for invasion assay according to the manufacturer's instructions. The assays were performed as described [20].

### 2.5. Quantitative Real-Time PCR (qRT-PCR)

Total RNAs were extracted by using TRIZOL reagent according to the manufacturer's protocol (Invitrogen, Carlsbad, CA, USA). 2 *μ*g RNAs were reversely transcribed by PrimeScriptTM RT reagent Kit (TaKaRa, Dalian, China). Quantitative PCR was performed by using SYBR Green PCR Master Mix (TaKaRa, Dalian, China) according to the manufacturer's instruction. GAPDH was used as an internal control, and 2^^-*ΔΔ*CT^ method was used for calculating the relative expression as described [21]. The primers used in the manuscript are shown in Table 1.

### 2.6. Western Blot Assay and Immunoprecipitation (IP)

Total protein was extracted from cells using RIPA Lysis Buffer (Thermo Scientific, USA) and quantified using BCA protein assay kit (Beyotime Biotechnology, China). Western blot and immunoprecipitation analysis were performed as described [22]. The antibodies for CD9 (sc-13118), CD81 (sc-166029), Clever-1 (sc-293254), *β*-actin (sc-8432), VEGF (sc-7269), and individual secondary antibodies were purchased from Santa Cruz (Santa Cruz Biotechnology, Santa Cruz, CA). The antibodies for HSP70 (#4873), Arg-1 (#93668), E-cadherin (#14472), N-cadherin (#13116), Vimentin (#5741), Snail (#3879), *β*-catenin (#8480), Ser33-phosphorylated *β*-catenin (#2009), c-Myc (#18583), GSK3*β* (#12456), and PCNA (#13110) were all obtained from Cell Signaling Technology (Cell Signaling Technology Inc, Beverly, USA). The working concentrations of antibodies were 1 : 1000 for all primary antibodies.

### 2.7. Animal Study and Immunofluorescence Experiment

Xenograft tumor experiments and *in vivo* lung metastasis models were approved by the Institutional Animal Care and Use Committee of Bengbu Medical College. The immunofluorescence experiments were performed as shown in previous study [23]. For lung metastasis model, 6 combined immunodeficient (SCID) mice at ~6 weeks old (Vital River, Beijing, China) were injected 1 × 10^6^ T24 cells via the tail vein. The mice were treated with M0 or TAM exosomes for 4 weeks and followed by the examination by H&E staining. For xenograft models, T24 cells (5 × 10^6^) were also subcutaneously injected into 5 mice followed by exosome treatment, and 4 weeks later, the mice were sacrificed and tumors were obtained.

### 2.8. Statistical Analysis

All data were presented as mean ± SD of three experiments. Two-tailed Student *t*-test was performed to determine the statistical significance, and *p* values < 0.05 were considered statistically significant.

## 3. Results

### 3.1. Identification of M2-Like TAMs from THP-1 and TAM-Derived Exosomes

**Figure 1:**
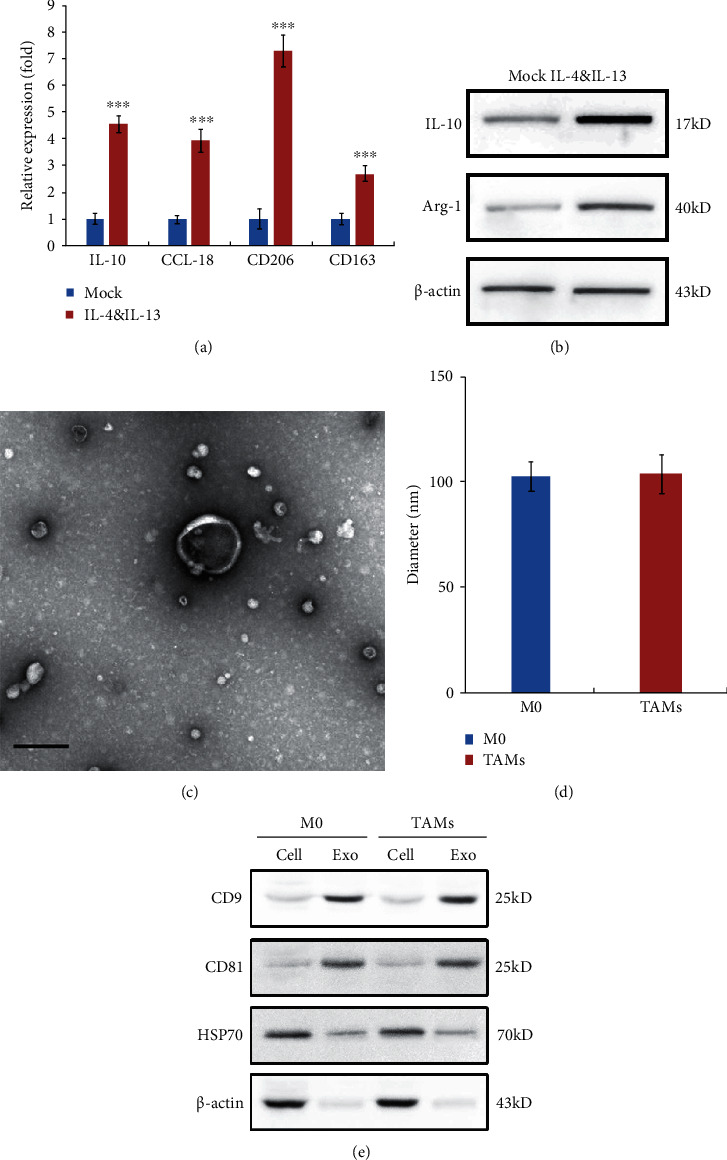
Identification of M2-like TAMs from THP-1 and TAM-derived exosomes. (a, b) M0 THP-1 cells were incubated with IL-4 and IL-13 to promote M2-TAM polarization, and the (a) mRNA levels of M2-TAM markers IL-10, CCL-18, CD206, and CD163 were detected by qRT-PCR, and the (b) protein levels of Clever-1 and Arg-1 (also M2-TAM markers) were examined by western blot. (c) Representative electron micrograph of exosomes derived from TAMs. Scale bar, 100 nm. (d) The diameter of exosomes derived from the medium of M0 or TAMs. (e) Western blot analysis of the expression of CD9, CD81, and HSP70 in the cells and exosomes. Data shown are representative images or expressed as the mean ± SD. ^∗∗∗^*p* < 0.001 compared to the control.

To illustrate the effect of TAM-derived exosomal lncRNA, we incubated M0 THP-1 cells with IL-4 and IL-13 to promote M2 polarization at first. The phenotype of M2-like TAMs was confirmed by qRT-PCR. As shown in Figure 1(a), the markers of M2-TAMs including IL-10, CCL-18, CD206, and CD163 were all increased by IL-4 and IL-13 administration. Consistently, protein levels of TAM markers Arg-1 and Clever-1 were also increased in IL-4 and IL-13 stimulated M0 THP-1 cells (Figure 1(b)). Moreover, we isolated exosomes from M0 and TAMs, and the identification was performed by transmission electron microscopy examination (Figure 1(c)), nanoparticle-tracking analysis (NTA) (Figure 1(d)), and western blot analysis of exosome marker proteins (Figure 1(e)). These results indicated that the THP-1 macrophages were polarized from M0- to M2-like TAM phenomenon, and the exosomes were purified from M0 and TAMs successfully.

### 3.2. TAM-Derived Exosome Promotes Migration, Invasion, and EMT Process of Bladder Cancer Cells

**Figure 2:**
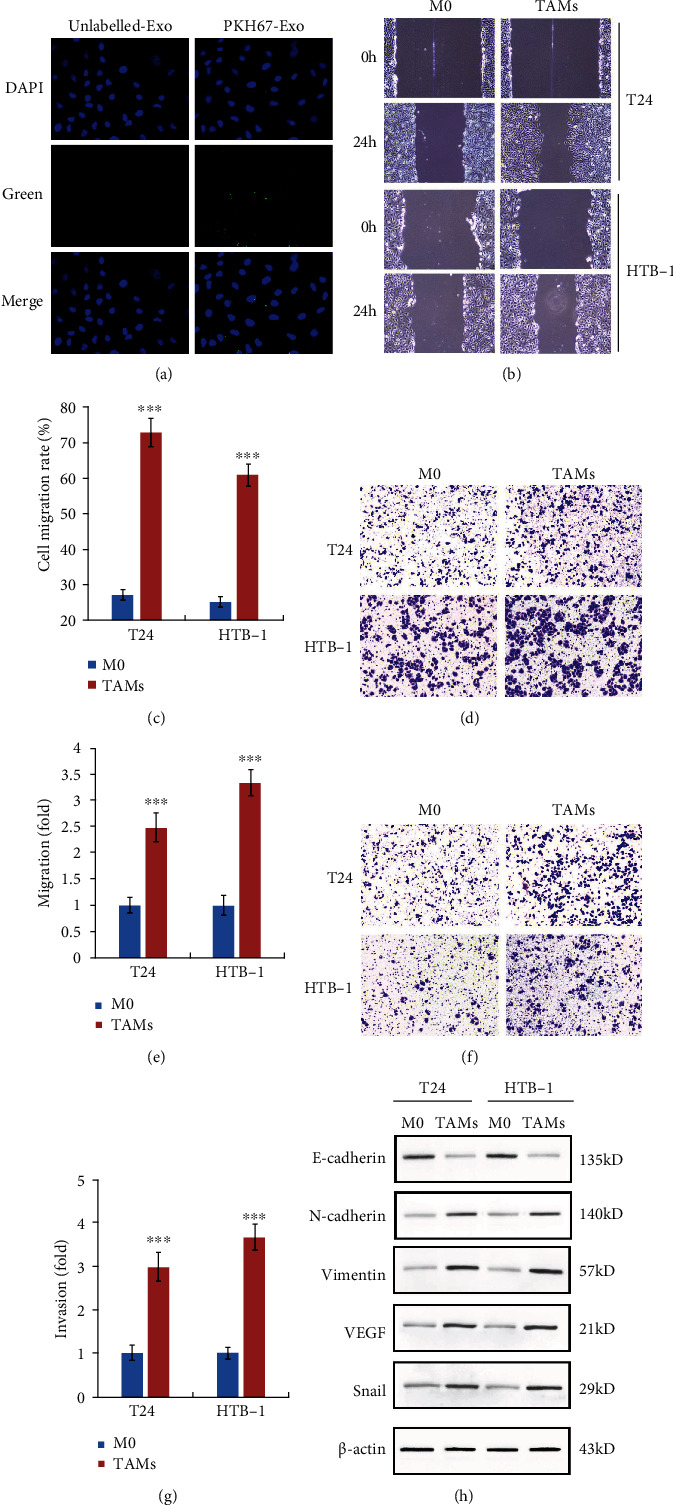
TAM-derived exosomes promotes migration, invasion, and EMT process of bladder cancer cells. (a) PKH67-labelled TAM exosomes were shown to be taken up by T24 BC cells. (b, c) Wound healing assay was performed to detect the effect of TAM-derived exosomes on migration of T24 and HTB-1 cells. (d, e) Transwell assay (without matrigel) was used to illustrate the effect of TAM-exo on cell migration. (f, g) Transwell assay (with matrigel) was used to illustrate the effect of TAM-exo on cell invasion. (h) EMT markers such as E-cadherin, N-cadherin, Vimentin, VEGF, and Snail were detected by western blot assay after TAM-exo treatment in T24 and HTB-1 cells. Data shown are representative images or expressed as the mean ± SD. ^∗∗∗^*p* < 0.001 compared to the control.

Polarized TAMs were cultured, and exosomes were isolated from the conditioned medium. To confirm that the exosomes from TAMs could be internalized by BC cells, T24 BC cells were incubated with PKH67-labelled exosomes derived from M2-TAMs. As shown in Figure 2(a), pKH67-labelled exosomes could be taken up by T24 BC cells. Due to the metastatic prone nature of BC, we examined the effect of TAM-exo in the metastasis and invasion processes. We found that TAM-exo administration obviously promotes T24 and HTB-1 cell metastasis abilities compared with M0-exo treatment (Figures 2(b) and 2(c)). Consistently, transwell assay results showed the enhanced metastasis effect of T24 cells and HTB-1 cells (Figures 2(d) and 2(e)). Furthermore, we found that the invasion abilities of T24 cells and HTB-1 cells were also aggravated by TAM-exo treatment (Figures 2(f) and 2(g)). In addition, we detected the markers of EMT process, and we found that in both T24 and HTB-1 cells, E-cadherin expression was decreased in TAM-exo-treated cells, and the expression of N-cadherin, Vimentin, VEGF, and Snail was increased upon TAM-exo administration compared with M0-exo treatment (Figure 2(h)). These results indicated that TAM-derived exosomes could be taken up by BC cells and subsequently promoted the metastasis, invasion, and EMT processes of BC cells.

### 3.3. lncRNA HISLA Expression Is Upregulated in TAM-Derived Exosomes

**Figure 3:**
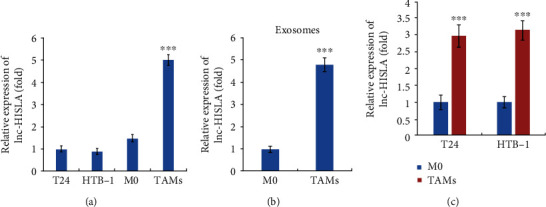
lncRNA HISLA expression is upregulated in TAM-derived exosomes. (a) The expression levels of lncRNA HISLA in T24, HTB-1, M0 THP-1, and M2-TAMs were examined by qRT-PCR. (b) Relative expression of lncRNA HISLA in exosomes derived from M0 THP-1 or M2-TAMs. (c) After treatment with M0-exo or TAM-exo, the relative levels of lncRNA HISLA in T24 or HTB-1 cells were detected by qRT-PCR. Data shown are representative images or expressed as the mean ± SD. ^∗∗∗^*p* < 0.001 compared to the control.

In order to investigate the role of lncRNA HISLA in BC, we detected the expression of lncRNA HISLA in T24, HTB-1, M0, and TAMs, and we found that lncRNA HISLA was significantly increased in M2-like TAMs (Figure 3(a)). Furthermore, we detected lncRNA HISLA levels in exosomes from M0 and TAMs, and we found that the expression of lncRNA HISLA was upregulated in TAM exosomes compared with M0 exosomes (Figure 3(b)). In addition, to confirm whether exosomal lncRNA HISLA derived from TAMs could be taken up by BC cells, we examined HISLA expression in TAMs or M0 exosome-treated T24 or HTB-1 cells, and we observed that both of these cells could internalized lncRNA HISLA therefore leading to the increased HISLA expression in BC cells (Figure 3(c)). These findings suggested that lncRNA HISLA expression is increased in TAM-derived exosomes, and lncRNA HISLA could be transferred from TAMs to BC cells.

### 3.4. Silencing of lncRNA-HISLA in TAM-exo Suppresses Migration, Invasion, and EMT Process of Bladder Cells

**Figure 4:**
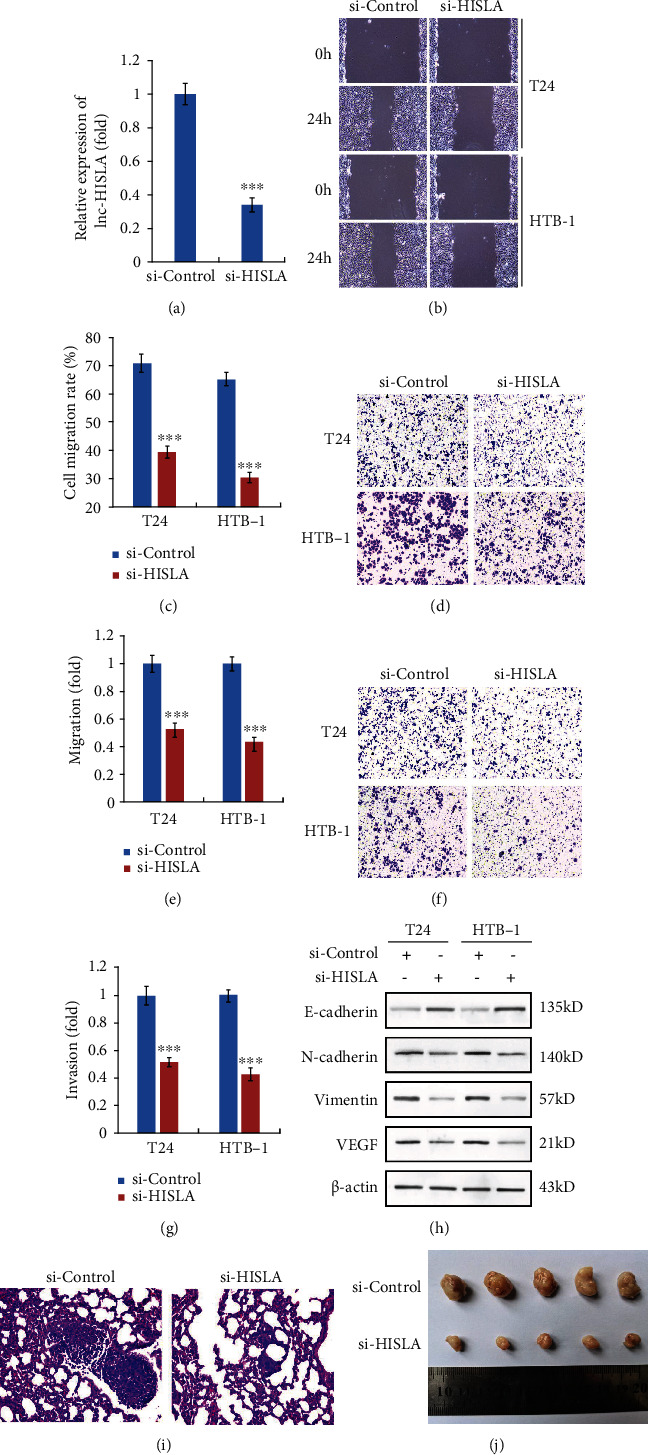
Silencing of lncRNA-HISLA in TAM-exo suppresses migration, invasion, and EMT process of bladder cells. (a) M2-TAMs were treated with si-Control or si-HISLA siRNAs, and the efficiency of silencing was confirmed by qRT-PCR. (b, c) Wound healing assay was performed to detect the effect of silencing HISLA in TAM-exo on migration of T24 and HTB-1 cells. (d, e) Transwell assay (without matrigel) was used to illustrate the effect of silencing HISLA in TAM-exo on cell migration. (f, g) Transwell assay (with matrigel) was used to illustrate the effect of silencing HISLA in TAM-exo on cell invasion. (h) EMT markers such as E-cadherin, N-cadherin, Vimentin, and VEGF were detected by western blot assay after TAM-exo treatment in T24 and HTB-1 cells. (i) Representative images of lung metastasis in metastatic experiments. (j) Representative photos of tumor tissues in xenograft models. Data shown are representative images or expressed as the mean ± SD. ^∗∗∗^*p* < 0.001 compared to the control.

Since we know the overexpression of lncRNA HISLA in exosomes from TAMs, we used siRNA-targeted HISLA to silence its expression and evaluate the precise effect of exosomal HISLA in BC cells. As shown in Figure 4(a), the efficiency of HISLA siRNA in TAM-exo was confirmed by qPCR. Moreover, T24 or HTB-1 cells were incubated with TAM exosomes transfected with control siRNA or HISLA siRNA, and the abilities of metastasis were significantly suppressed in si-HISLA exosome-treated BC cells compared with the si-Control groups (Figures 4(b) and 4(c)). Consistent with the wound healing assay results, transwell data also indicated that metastasis ability was inhibited in BC cells incubated with HISLA-silenced TAM exosomes (Figures 4(d) and 4(e)). Similar results of invasion ability were observed in BC cells after HISLA siRNA exosome treatment (Figures 4(f) and 4(g)). In addition, lncRNA HISLA siRNA-treated exosomes led to the increased expression of E-cadherin and downregulation of N-cadherin, Vimentin, and VEGF, which meant that silencing of HISLA in TAM-exo suppressed the EMT potential of BC cells (Figure 4(h)). Furthermore, we performed *in vivo* metastatic and xenograft experiments, and we found that silencing of HISLA significantly alleviated lung metastasis of tumor cells and suppressed tumor development (Figures 4(i) and 4(j)).

### 3.5. Exosomal lncRNA-HISLA from TAMs Promotes Wnt/*β*-Catenin Signaling Activation in Bladder Cells

**Figure 5:**
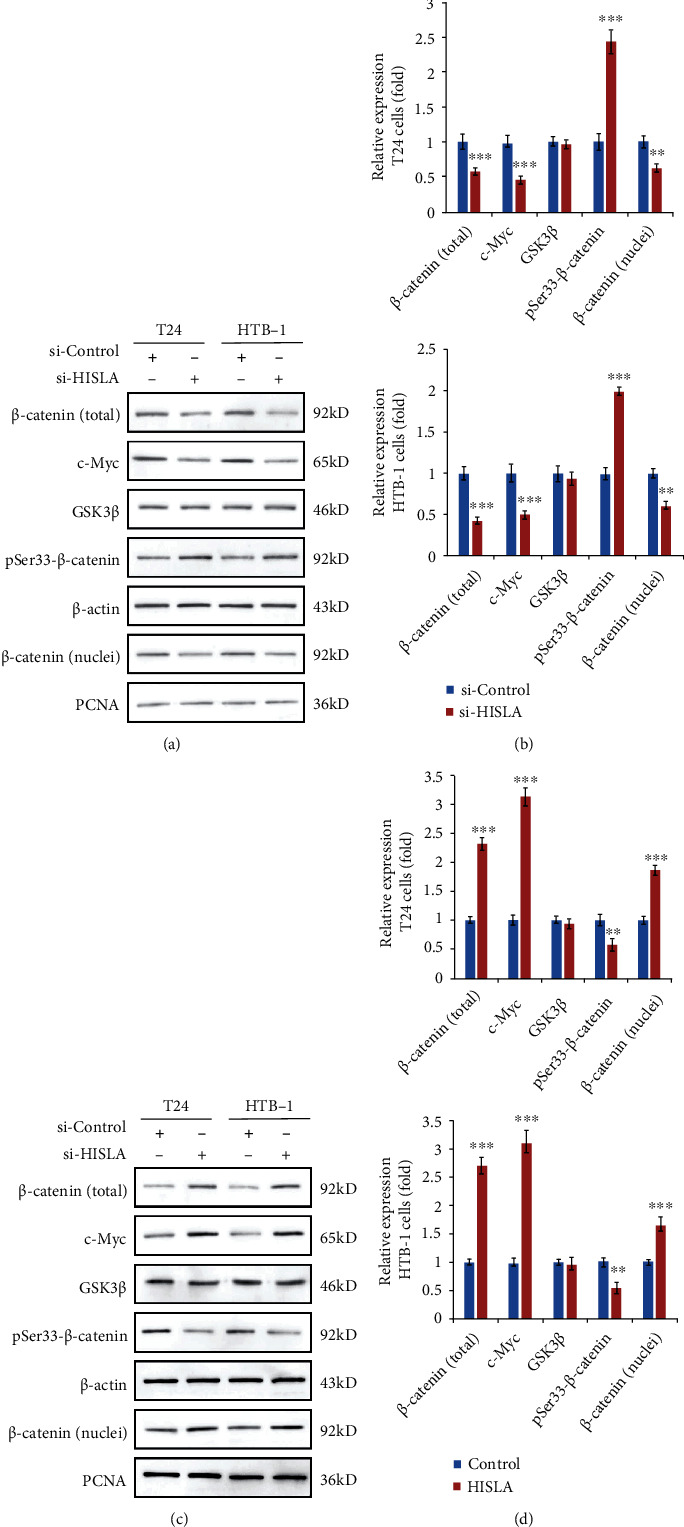
Exosomal lncRNA-HISLA from TAMs promotes Wnt/*β*-catenin signaling activation in bladder cells. (a, b) Protein levels of *β*-catenin (total), *β*-catenin (nuclei), c-Myc, GSK3*β*, and pSer33-*β*-catenin in HISLA silenced TAM-exosome-treated BC cells. (c, d) Protein levels of *β*-catenin (total), *β*-catenin (nuclei), c-Myc, GSK3*β*, and pSer33-*β*-catenin in HISLA overexpressed TAM-exosome-treated BC cells. Data shown are representative images or expressed as the mean ± SD. ^∗∗∗^*p* < 0.001 compared to the control.

Wnt/*β*-catenin signaling pathway has been proved to play crucial roles in the regulation of bladder cancer progression and contributes to EMT process [24–26]; therefore, we investigated whether TAM exosomal lncRNA HISLA could regulate EMT ability of BC cells through Wnt/*β*-catenin signaling. As shown in Figures 5(a) and 5(b), silencing of HISLA in TAM-exo led to the decreased expression of total *β*-catenin, nuclei *β*-catenin, and downstream c-Myc in both T24 and HTB-1 cells. Moreover, the levels of Ser33-phosphorylated *β*-catenin which lead to the proteasomal degradation of *β*-catenin were significantly increased in BC cells treated with HISLA-silenced TAMs-exo, while the expression of GSK3*β* which is responsible for Ser33-phosphorylation of *β*-catenin was not affected during si-HISLA or si-Control TAM-exo-treated BC cells (Figures 5(a) and 5(b)). Consistent results of HISLA overexpressed TAM-exo-treated BC cells were observed as shown in Figures 5(c) and 5(d) that overexpression of HISLA in exosomes derived from TAMs conferred to the increased *β*-catenin expression and increased activation of *β*-catenin. These findings indicated that TAM-derived exosomal lncRNA HISLA promoted the activation of Wnt/*β*-catenin signaling pathway.

### 3.6. Exosomal lncRNA-HISLA Derived from TAMs Stabilized *β*-Catenin in BC Cells through Preventing Interaction between GSK3*β* and *β*-Catenin

**Figure 6:**
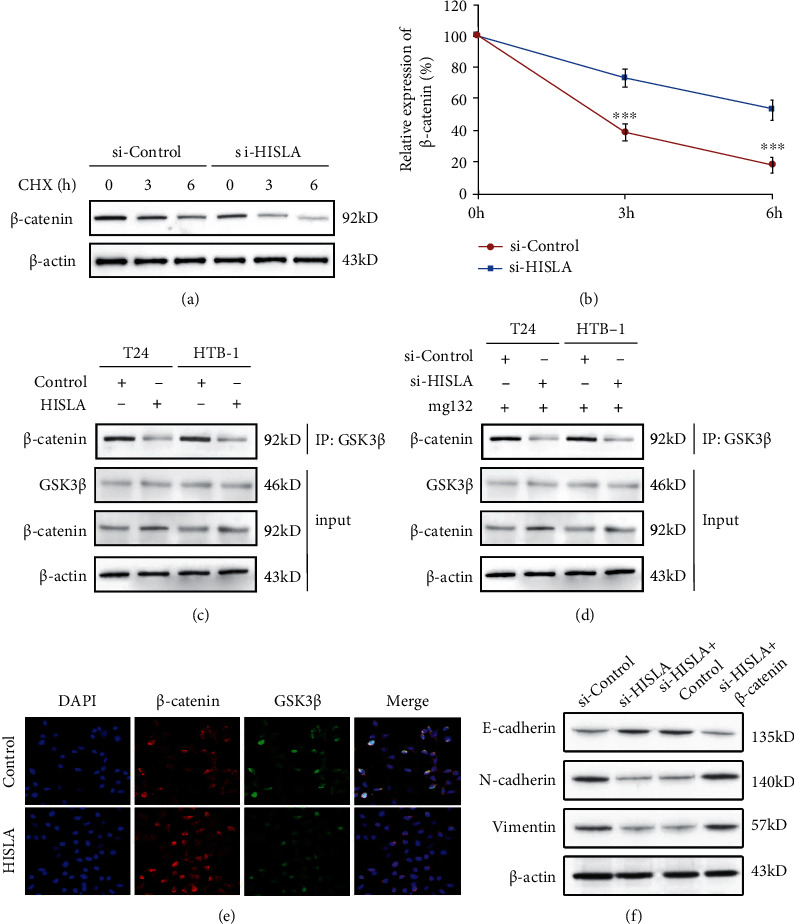
Exosomal lncRNA-HISLA derived from TAMs stabilized *β*-catenin in BC cells through preventing interaction between GSK3*β* and *β*-catenin. (a, b) After treatment with protein synthesis inhibitor CHX for indicated times, the protein levels of *β*-catenin in HISLA silenced TAM-exosome-treated T24 cells were examined by western blot. (c) Interaction between GSK3*β* and *β*-catenin was detected by immunoprecipitation in HISLA overexpressed T24 or HTB-1 cells. (d) Interaction between GSK3*β* and *β*-catenin was detected by immunoprecipitation in HISLA silenced T24 or HTB-1 cells with the existence of proteasome inhibitor mg132. (e) Immunofluorescence was used to analyze the interaction between GSK3*β* and *β*-catenin in HISLA overexpressed T24 cells. (f) si-HISLA and *β*-catenin were cotransfected into T24 cells, and the protein levels of E-cadherin, N-cadherin, and Vimentin were detected by western blot. Data shown are representative images or expressed as the mean ± SD. ^∗∗∗^*p* < 0.001 compared to the control.

Cycloheximide (CHX) was always used for the analysis of protein half-life; therefore, we used CHX to examine the effect of HISLA on *β*-catenin stabilization. We found that silencing of HISLA in TAM-exo obviously enhanced the degradation of *β*-catenin (Figures 6(a) and 6(b)). GSK3*β* was well-known to interact with *β*-catenin and promote the phosphorylation of *β*-catenin at Ser33, Ser37, and Thr41, therefore to promote the proteasomal degradation of *β*-catenin [27]. Therefore, we investigated whether lncRNA HISLA contributed to the interaction between GSK3*β* and *β*-catenin. Interestingly, we found that overexpression of HISLA in TAM-exo significantly interrupted the interaction between GSK3*β* and *β*-catenin (Figure 6(c)), while silencing of HISLA in exosome derived from TAMs enhanced the interaction among GSK3*β* and *β*-catenin (Figure 6(d)) with the existence of mg132 which was a widely used proteasome inhibitor. Furthermore, we performed immunofluorescence experiment, and we found that overexpression of HISLA significantly inhibited the interaction between GSK3*β* and *β*-catenin (Figure 6(e)). By using rescue experiment, we confirmed that HISLA regulated EMT potential mainly through *β*-catenin signaling (Figure 6(f)). These data revealed that exosomal lncRNA derived from TAMs inhibited the interaction between GSK3*β* and *β*-catenin, therefore suppressed the phosphorylation of *β*-catenin, and the stabilized *β*-catenin led to the abnormal activation of EMT process and promotion of metastasis and invasion of BC cells.

## 4. Discussion

In the current study, we illustrated the role of TAM exosome-derived lncRNA HISLA in metastasis, invasion abilities, and EMT potential of BC cells. To the best of our knowledge, this manuscript is the first publication about the relationship between TAM-derived exosomal lncRNA HISLA and bladder cancer.

Tumor associated macrophages (TAMs) are macrophages infiltrating into tumor tissue and an important component of tumor microenvironment (TME), and the abundance of TAMs in tumor tissues is always correlated with poor prognosis of solid tumors usually [28]. In bladder cancer, TAM has been found to be closely related to the progression of bladder cancer. For instance, M2-like TAMs driven by specific genomic alterations were reported to be associated with prognosis in bladder cancer [29]. M2-like TAM infiltration in TME also observed to increase the expression of CXCL8, which promoted the cancer progression by affecting migration and invasion processes of bladder cells [30]. In turn, tumor cells will also enhance their own development by promoting M2 polarization. For example, previous study showed that BMP4 ligands of bladder cells induced M2-like TAM polarization and favored bladder cancer progression [31]. Bladder cancer cells could also secrete exosomal miR-21 to promote cancer development by promoting activation of STAT3-induced M2 polarization [32]. LINC01140 in bladder cells were found to modulate macrophage M2-TAM polarization and aggravated aggressiveness of bladder cancer cell [33]. In the current study, we investigated the effect of TAM-derived exosomes in bladder cancer progression. The M0 THP-1 cells were incubated with IL-4 and IL-13 to induce M2-TAM phenotype, and the expression of markers of M2-TAMs such as IL-10, CCL-18, CD206, CD163, Clever-1, and Arg-1 was detected and confirmed. Furthermore, we found that TAM-exo treatment significantly promoted the migration, invasion, and EMT potential of bladder cells compared with M0 exosome administration, which indicated that TAM infiltration may play crucial roles in bladder cell progression.

Long noncoding RNA (lncRNA) often refers to noncoding RNA with a length of more than 200 nucleotides which has been proved to be closely correlated with tumorigenesis and development [34–36]. At present, amounts of lncRNAs have been shown to contribute to nearly all the processes of bladder cancer. For instance, lncRNA-RMRP sponged miR-206 to promote proliferation, migration, and invasion of bladder cancer [37]. lncRNA CASC11 promoted bladder cancer cell proliferation through miRNA-150 [38]. lncRNA GClnc1 was found to promote proliferation and invasion of bladder cancer via activation of Myc [39]. lncRNA-SLC16A1-AS1 has been shown to induce bladder cancer metabolic reprogramming as target and coactivator of E2F1 [40]. lncRNA HIF-1*α*-stabilizing long noncoding RNA (HISLA) has been reported to suppress the interaction of PHD2 and HIF-1*α* to inhibit the hydroxylation and degradation of HIF-1*α*, leading to the regulation of aerobic glycolysis of breast cancer cells [41]. Serum HISLA expression in breast cancer patients was significantly increased compared to the healthy controls, which suggested lncRNA HISLA as a potential biomarker for breast cancer diagnosis and prognosis [42]. However, the role of lncRNA HILSA in bladder cancer still remains largely unknown. In the current research, we revealed that lncRNA HISLA from TAM-exo could be taken up by bladder cancer cells, and silencing of HILSA in TAM-exo alleviated the metastasis, invasion, and EMT marker expression of bladder cancer cells, which indicated that exosomal lncRNA HISLA derived from TAMs promoted EMT potential of bladder cancer cell.

Epithelial-mesenchymal transition (EMT) refers to the biological process of epithelial cells transforming into motile mesenchymal cells with interstitial phenotype through specific procedures, and it plays critical roles in embryonic development, chronic inflammation, tissue reconstruction, and cancer metastasis [43]. Wnt/*β*-catenin is well-known signaling pathway regulating EMT process and has been proved to be essential for the migration and invasion of various kinds of cancer cells [44]. During want/*β*-catenin-induced activation of EMT, the regulation of *β*-catenin expression is one of the most important limiting steps to control the procedure. *β*-Catenin is successively phosphorylated by CKI and GSK-3*β*, followed by reorganization by *β*-Trcp and ubiquitination by an E3 ubiquitin ligase complex, which lead to the proteasomal degradation of *β*-catenin [45]; therefore, the regulation of GSK-3*β*-induced *β*-catenin phosphorylation is crucial for the EMT activation. In this research, we found that exosomal lncRNA HISLA positive correlated with the expression of *β*-catenin, and negative correlation was found between HISLA expression and *β*-catenin Ser33 phosphorylation in BC cells. In addition, exosomal lncRNA HISLA derived from M2-like TAMs was found to stabilize *β*-catenin through interrupting the interaction between GSK3*β* and *β*-catenin and finally led to the overactivation of EMT abilities of BC cells.

## 5. Conclusion

In the current study, we revealed the bladder cancer promoting effect of exosomal lncRNA HISLA derived from M2-like TAMs by regulating *β*-catenin-induced EMT activation and suggested lncRNA HISLA from TAM exosomes as a potential medical target for the bladder cancer treatment.

## Figures and Tables

**Table 1:** List of primers used in the manuscript.

Number	Gene		Sequence (5′-3′)
1	IL-10	F	TGC CTA ACA TGC TTC GAG ATC
		R	CCA GGT AAC CCT TAA AGT CCT C
2	CCL-18	F	GTT GAC TAT TCT GAA ACC AGC CC
		R	GTC GCT GAT GTA TTT CTG GAC CC
3	CD206	F	AGC CAA CAC CAG CTC CTC AAG A
		R	CAA AAC GCT CGC GCA TTG TCC A
4	CD163	F	CCA GAA GGA ACT TGT AGC CAC AG
		R	CAG GCA CCA AGC GTT TTG AGC T
6	HISLA	F	TGA GTA GAA GAG AGT GGG GAG GG
		R	ACT GTG GCA TGG TGA TTG TTT GG
6	GAPDH	F	CTG GGC TAC ACT GAG CAC C
		R	AAG TGG TCG TTG AGG GCA ATG

## Data Availability

All data were available upon requesting from the corresponding author.
