# Correction to “M2 Tumor Associate Macrophage- (TAM-) Derived lncRNA HISLA Promotes EMT Potential in Bladder Cancer”

**DOI:** 10.1155/jo/9754691

**Published:** 2025-10-10

Y. Guo, Z. Li, W. Sun, et al., “M2 Tumor Associate Macrophage‐(TAM‐) Derived lncRNA HISLA Promotes EMT Potential in Bladder Cancer,” *Journal of Oncology* 2022 (2022): 8268719, https://doi.org/10.1155/2022/8268719.

In the Results section, Figure 6(d) is incorrect. During the preparation of the manuscript, Figure 6(c) was mistakenly uploaded in duplicate. The correct Figure 6 is shown below.


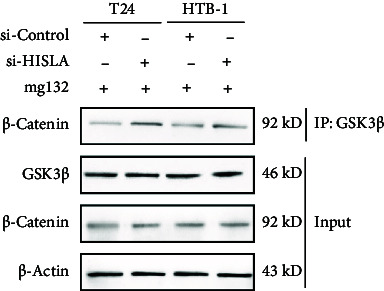


We apologize for this error.

